# Major Improvements of Quartz Crystal Pulling Sensitivity and Linearity Using Series Reactance

**DOI:** 10.3390/s91008263

**Published:** 2009-10-19

**Authors:** Vojko Matko, Riko Šafarič

**Affiliations:** University of Maribor, Faculty of Electrical Engineering and Computer Science, Smetanova 17, 2000 Maribor, Slovenia; E-Mail: riko.safaric@uni-mb.si

**Keywords:** quartz crystal, pulling range, pulling linearity

## Abstract

This paper presents a new method of substantially improving frequency pullability and linearity using reactance in series with an AT fundamental crystal operated with a series load capacitance in the range of 3 to 50 pF and frequencies in the range of 3.5 to 21 MHz. The research describes high quartz pullability and linearity by varying the load capacitance. The paper also gives impedance circuits for crystal unit (3.5 MHz) together with load capacitance and compensation reactance. The experimental results show that the new approach using compensation method of quartz crystal connected in series reactance increases the frequency pulling range by ×25 to ×100 depending on the type of oscillator and compensation factor “k” in the temperature range of 10 to 40 °C.

## 1. Introduction

Quartz crystals are generally suited for the manufacture of frequency selection or frequency control devices. In oscillators with load capacitance in series with the crystal unit, the oscillation frequency depends on the capacitive load that is applied. The amount of nonlinear frequency change as a function of load capacitance is referred to as the pullability. It indicates how far from the nominal frequency (intended oscillating frequency) the resonant frequency can be forced by applying the load [1]. Typically, it is used to tune the operating frequency to a desired value. In special cases, it can also be used for the measurement purposes, allowing the measurement of various quantities based on capacitive influence on the quartz crystal oscillation frequency [2-3]. However, when these various quantities are measured, the problem of insufficient sensitivity and nonlinear characteristics very often arises.

This research focuses on the pulling sensitivity and linearity of the AT fundamental quartz crystals (cut angle: +2′) operating over the measurement temperature range of 10 to 40 °C. Crystals fabricated in this manner exhibit excellent frequency *vs.* temperature stability. They have fundamental resonant frequencies between 3.5 and 21 MHz.

## 2. Compensation of C_o_ and Pulling Sensitivity Improvement

The equivalent circuit is an electrical representation of the quartz crystal's mechanical and electrical behaviour. The components C, L, and R are called the motional arm and represent the mechanical behaviour of the crystal element. C_o_ represents the electrical behaviour of the crystal element and holder. Typical quartz data of 3.5 MHz resonance frequency (fundamental mode) is as follows: fr = 3.5 MHz, C = 25 fF, L = 82.8 mH, R = 10 Ω and C_o_ = 4 pF. The values in the quartz crystal equivalent circuit were measured by a HP 4194A impedance/gain-phase analyzer.

The capacitance C_o_ is a real capacitance, comprising the capacitance between the electrodes and the stray capacitance associated with the mounting structure. It is also known as the “shunt” or “static” capacitance, and represents the crystal in a non-operational, or static state. Depending on the particular enclosure type, C_o_ normally lies between 1 and 7 pF. Oscillator crystals are normally designed with C_o_ less than 7 pF. One possibility how to increase the pulling sensitivity is to reduce C_o_ in Equation 1, which is the serial resonant frequency for the crystal in series with load capacitence C_z_ [1]:

(1) $$\text{fs}(\text{Cz}):=\frac{\sqrt{1+\frac{\text{C}}{\text{Co}+\text{Cz}}}}{2\cdot \pi \sqrt{\text{L}\cdot \text{C}}}$$

The other possibility is to compensate C_o_ with parallel inductance L_p_ connected to basic quartz crystal equivalent circuit providing that ω_o_·L_p_ = 1/ (ω_o_·C_o_), resulting in Equation 2 [4-7]:

(2) $$\text{fsp}(\text{Cz}):=\frac{\sqrt{1+\frac{\text{C}}{\text{Cz}}}}{2\cdot \pi \sqrt{\text{L}\cdot \text{C}}}$$

**Figure 1.**
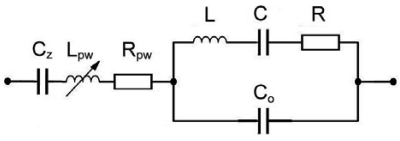
Load capacitance C_z_ and compensation impedance Z_pw_ = j·ω·L_pw_ + R_pw_ in series with the quartz crystal equivalent circuit.

The novelty lies in the compensation of C_o_ with inductance L_pw_ providing that ω_o_·L_pw_ = 1/ (ω_o_·C_o_) (Figure 1) with the criterion k·L_pw_ = 1/ k·C_o_. Resistance R_pw_ is a real part of impedance Z_pw_.

**Figure 2.**
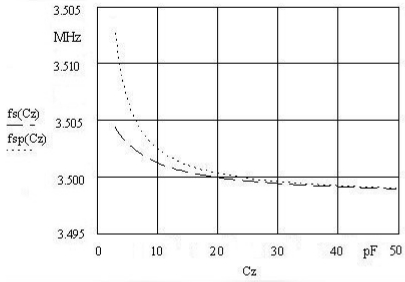
Quartz crystal pulling sensitivity from 3 to 50 pF.

Figure 2 shows a comparison of the pulling sensitivity between Equations 1 and 2 for a 3.5 MHz quartz crystal in the capacitance range of 3–50 pF. The change of frequency is approximately two times higher if we compensate C_o_ [dependence f_sp_(C_z_)]. For the general sensitivity measurement purposes, the capacitance range between 3 pF and 20 pF is the most useful. It is in this range that the frequency capacitance dependence is the greatest (the highest pulling sensitivity). The highest frequency sensitivity is in the range 3–10 pF, where a very small capacitance changes can be measured (aF range). Figure 2 also shows typical nonlinear frequency dependence and a small pulling range [dfs(Cz) ∼ 40 kHz].

## 3. The Use of Serial Reactance for Linearity and Pulling Sensitivity Improvement

Taking into account that:

(3) $$\text{fr}:=\frac{1}{2\cdot \pi \sqrt{\text{L}\cdot \text{C}}}$$

where -f_r_ is resonant frequency with phase 0, and

(4) $$\omega_{0}:=2\cdot \pi \cdot {\text{f}}_{\text{r}}$$

using the inductance L_pw_ connected in series and providing that k·L_pw_ = 1/ k·C_o_ (Equation 5),

(5) $$\text{Lpw}:=\frac{1}{\omega {\text{o}}^{2}\cdot \text{Co}}$$

we get Equation 6, where we have a linearized frequency dependance with regard to C_z_ for various C_o_ values which change with “k” values. This represents a novelty in this research. Due to very small inductance L_pw_, the real resistance R_pw_ can be ignored (Figure 1) [4].

k = 1, 2, 3

(6) $$\text{fsk}(\text{Cz}):=\frac{1+\frac{\text{C}}{2\cdot \left(\frac{1}{k}\cdot \text{Co}-\frac{1}{\omega 0^{2}\cdot \text{k}\cdot \text{Lpw}-\frac{1}{\text{Cz}}}\right)}}{2\cdot \pi \sqrt{\text{L}\cdot \text{C}}}$$

Table 12 shows typical quartz data and experimental pulling results (dfs, dfsk for k = 1 and k = 2) for four different quartz crystals in the frequency range 3.5 to 21 MHz. The novelty here is in the pullability increase (dfsk) at values k = 1 in k = 2 (Equation 6), taking into account Equation 5. The values in the quartz crystal equivalent circuit were measured by the HP 4194A impedance/gain-phase analyzer.

For the given various frequencies the quartz crystal data could also be different (C_0_, C, R, L). The frequency changes of dfs and dfsk (for k = 1 and 2) are measured at various C_z_ values (3 pF and 50 pF).

## 4. Quartz Frequency Stability

The maximum attainable stability of a crystal unit is dependent on the high Q value (3.5 MHz – Table 2). The smaller the distance between f_r_ (series resonant frequency) and f_p_ (parallel resonant frequency) the higher the Q value, and the steeper the slope of the reactance. The factors that further limit the Q are mounting loss, atmospheric loading (for non-evacuated crystal units) and the surface finish of the blank. Mounting loss depends upon the degree of trapping produced by the electrode and the plate diameter. The highest Q of quartz unit is important because of the frequency stability:

(7) $$\text{Q}:=\frac{\omega \text{o}\cdot \text{L}}{\text{R}}$$

The figure of merit is a useful indicator, particularly for oscillator application and shows as the difference between f_r_ and f_s_ (pulling frequency difference value). In an oscillator, for a resonator with M less than 2, the sustaining circuits must present inductive impedance to the crystal unit. As M increases beyond 2, f_r_ and f_a_ separate and, for large M, approach f_s_ and f_p_, respectively. In general, the larger M is the more useful resonator (the greater crystal oscillation stability):

(8) $$\text{M}:=\frac{1}{\omega \text{o}\cdot \text{R}\cdot \text{Co}}$$

As a consequence of hysteresis, the frequency *vs.* temperature curves obtained by slowly increasing the temperature from, say, 10 °C to 40 °C will not coincide with the curve obtained by slowly decreasing the temperature from 40 °C to 10 °C. Frequency stability also depends on the temperature coefficient of the core material used of L_pw_. The proper choice of the core material is also the key in the sense of the frequency stability.

In general, the oscillator's circuit long-term stability also depends upon the crystal aging. Cold weld packages which are specially processed and welded in a high vacuum offer much better ageing rates and typically the ageing rates of cold weld crystal is less than ±1 ppm/year (10 °C to 40 °C). Stability of the electronic circuit depends upon the circuit type and quality of its elements [8-11]. The frequency stability ±0.1 Hz can be achieved provided that the above mentioned facts are taken into account and that the oscillator circuit is selected appropriately [1]. Another very important criterion for oscillator application is the drive level, which may not exceed 20 μW [12].

## 5. Experimental Results

**Figure 3.**
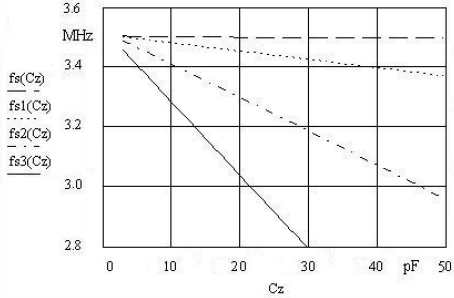
Quartz crystal pulling and linearization for k = 1, 2, 3 in the range C_z_ = 3–50 pF.

By satisfying the conditions of Equation 5, Equation 6 may be written, considerably increasing the frequency pulling range and linearizing it as shown in Figure 3 for the experimental data for the typical quartz data of 3.5 MHz resonance frequency (fundamental mode).

Figure 3 shows linear dependence for various k values as well as increased pulling (depending on the values of C_o_ and L_pw_ [Equation (6)] and also shows frequency dependences for serial resonant frequency fs(C_z_) (without compensation), dependance of function fs1(C_z_) (with compensation) for k = 1, fs2(C_z_) for k = 2 and fs3(C_z_) for k = 3, and the last one being very hard to achieve due to a very small capacitance C_o_ ≅ 1.33 pF. The oscillator frequency measurement error is approximately ±0.1 Hz [1].

We can define pulling sensitivity S as the frequency change in parts per million per pF change at a given load capacitance C_z_ for various k:

(9) $$\text{S}:=\frac{\text{C}}{2\cdot {\left(\frac{1}{\text{k}}\text{Co}+\text{Cz}\right)}^{2}}$$

In such a way, we can determine “S” for 3 to 50 pF. Since C_o_ and C are the same throughout our experiments (Table 1), we get the same “S” values for different k values (Table 3) for the frequencies 3.5 to 21 MHz (Table 2).

If we define the frequency ratio Ω = ω/ω_0_, which depends on $\omega_{0}=1/\sqrt{L\cdot C}$, and taking into account ω_0_L = 1/ω_0_C, the impedance equation for a crystal unit with C_z_ and L_pw_ is [1], [13]:

Ω = 0.998, 0.99802…1.038.

(10) $$\text{Zk}(\Omega ):=\left[\text{R}\cdot \frac{1+\omega \text{o}\cdot \left(\frac{\text{L}}{\text{R}}\right)\cdot \left(\Omega -\frac{1}{\Omega }\right)\cdot \text{j}}{1+\frac{\text{Co}}{\text{k}\cdot \text{C}}\cdot (1-\Omega^{2})+\frac{\text{Co}}{\text{k}\cdot \text{C}}\cdot \frac{\text{R}}{\omega \text{o}\cdot \text{L}}\cdot \Omega \cdot \text{j}}\right]+\left(\frac{\Omega \cdot 2\cdot \pi \cdot \text{k}\cdot \text{Lpw}\cdot \text{j}}{\sqrt{\text{L}\cdot \text{C}}}\right)+\text{Rpw}+\frac{\sqrt{\text{L}\cdot \text{C}}}{\Omega \cdot 2\cdot \pi \cdot \text{Cz}\cdot \text{j}}$$

**Figure 4.**
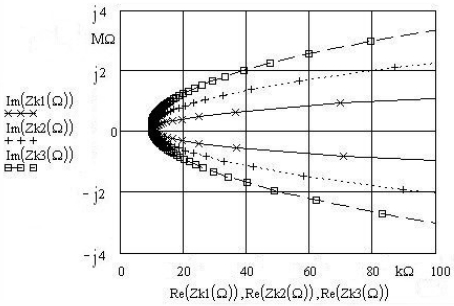
Compensated quartz impedance (C_o_) for different k = 1, 2, 3 (Ω = 0.998, 0.99802…1.038).

At the frequency of 3.5 MHz and data for C_z_ = 3 × 10^-12^ and k = 1, 2, 3 we get the three complex impedances as shown on Figure 4 (Equation 10). Ω, representing the change of resonant frequency in the vicinity of the serial resonant frequency f_r_ (the point where Im(Z_k_) = 0 represents the series resonant frequency f_r_). Figure 4 shows that by increasing the “k” values, Im(Z_k_(Ω)) and the pulling sensitivity of the quartz crystal increase as well as illustrated on Figure 3 (at value k = 3 the complex value of impedance Im(Zk3(Ω)) is doubled).

## 5. Conclusions

Experimental results of the comparison between compensated quartz crystal equivalent circuit and those using a non-compensated quartz crystal equivalent circuit show that the use of series reactance compensated crystals of the same frequency increases the pulling range by ×25 for k = 2 and ×100 for k = 3 also depending on the circuit used. It is the increase of pulling and a simultaneous linearilization that represents a novelty and a major advantage of this method in the measurement of femto and atofarad ranges. When the load capacitance is connected in series with the crystal, the frequency of operation of the oscillator is linearly increased inside limited values. It should also be emphasized that the exact pulling limits depend on the crystal's Q-value as well as associated stray capacitances. The most common factors affecting frequency stability such as operating temperature range, aging, hysteresis and drive level as well as all other crystal characteristics influencing the stability should also be considered because a stable oscillator circuit plays an important role in the increase of pulling and linear frequency dependance. Increased pulling range obtained experimentally can be used for determination of many different measurements such as strain, compression, positioning, angle, level, pressure, humidity, dielectric, biological growth, bacteria growth, and many other non-electrical quantities [14-16].

## Figures and Tables

**Table 1.** Quartz data and pulling sensitivity in frequency range 3.5 MHz to 21 MHz.

									
									Pulling sensitivity measured between C_z_ = 3 and 50 pF	
									
									k = 1	k = 2

f_r_ (MHz)	R(Ohm)	C(fF)	L(mH)	C_o_(pF)	L_pw_(uH)	Q (k)	M	dfs(kHz)	dfsk(kHz)	dfsk(MHz)
3.5	10	25	82.83	4	520.0	181.98	1,137	5,431	128	0.514
9	10	25	12.53	4	78.2	70.68	442	13,979	330	1,322
15	10	25	4.46	4	28.1	42.03	266	23,402	553	2,214
21	10	25	2.30	4	14.4	30.34	188	32,589	771	3,083

**Table 2.** Quartz data and pulling sensitivity S.

	**C_z_ = 3 pF**			**C_z_ = 50 pF**		
	k = 1	k = 2	k = 3	k = 1	k = 2	k = 3
S	2.551 × 10^8^	5 × 10^8^	6.657 × 10^8^	4.287 × 10^8^	4.663 × 10^8^	4.744 × 10^8^
